# Supplementary material for: Incidence of and trends in hip fracture among adults in urban China: A nationwide retrospective cohort study
Source: PLoS Med. 2020 Aug 6;17(8):e1003180. doi: 10.1371/journal.pmed.1003180 (PMC7410202; doi:10.1371/journal.pmed.1003180)
Supplement: S1 Appendix — (DOCX) [file pmed.1003180.s001.docx]

**S1 Appendix Statistical Methods and Analysis Plan**

1. **Case identification.**

Texts containing hip fracture diagnostic information were extracted from the medical insurance databases. To avoid missing patients when using the medical terms in Chinese, a fuzzy string matching algorithm was used to extract potential hip fracture patients from the databases. Keywords were first defined according to ICD-9 code, ICD-10 codes and medical terms in Chinese, shown in **Table A1**. The ICD codes were selected following previous publications [1-4]. The search algorithm was therefore defined as:

**(820 OR S72.0 OR S72.1).* OR .*(股骨颈.*骨折) .* OR .* (粗隆间.*骨折) .* OR .* (转子间.*骨折) .* OR .* (髋部.*骨折) .* OR .* (髋部疼痛) .* OR .* (下肢短缩外旋畸形) .* OR .* (髋部X线) .* OR .* (股骨颈骨折切开复位内固定) .* OR .* (股骨粗隆间骨折切开复位内固定).*

Second, based on the searching algorithm, two orthopedic surgeons performed manual verification of the diagnostic text with the assistance of the computer algorithm. The flow-chart for the case ascertainment was shown in **Figure A1**. The inclusion criteria for targeted cases was defined as patients with (1) femoral neck fracture; (2) intertrochanteric fracture; (3) hip fracture; (4) ICD-9 code 820; or (5) ICD-10 codes S72.0 and 72.1. The exclusion criteria for case identification were the following (1) pathological fracture; (2) old hip fracture; (3) femoral shaft fracture; (4) distal femoral fracture; (5) subtrochanteric fracture; (6) complications and sequelae of hip fracture (non-union; delayed union; malunion; osteomyelitis; osteoarthritis and anchylosis); (7) prosthesis complications; (8) osteonecrosis of the femoral head; (9) hip dislocation; or (10) removal of internal fixation devices. Patients were not included as a new case if (a) the fracture occurred within half a year after a previous one (this strategy is consistent with previous publications [5]); or (b) the diagnosis text was clearly described as “old hip fracture” by doctors.

As shown in **Figure A1**, in the verification stage, first of all, we generated the codebook for tagging hip fractures. Total 914,056 unique expressions were obtained using the searching algorithms defined above from both UEBMI and URBMI. These expressions were then segmented using Jieba Chinese word segmentation tool to gain unique diagnostic terms, according to a clinical terminology database. Overall, 320,367 terms were obtained. Then, a cosine distance function, shown as equation A1, was defined to measure the similarity between the diagnostic term and standard definition for hip fracture case (i.e. inclusion/exclusion of target patients defined above).

$$\sin\left( X,Y \right)=\cos\theta=\frac{\vec{x}\cdot\vec{y}}{\left\| x \right\|\cdot\left\| y \right\|} (A1)$$

where X and Y represented the vector of query and matching word respectively.

A threshold of 0.6 was set to create the term list similar to the regular expression of hip fracture. This term list was then mapped back to the corresponding diagnostic text or codes. These diagnostic text or codes were provided to two orthopedic surgeons independently for manual review. After that, a codebook of hip fracture related diagnosis texts and/or codes were summarized for tagging.

Third, we used the summarized tagging codebook to classify the records based on the searching algorithm from both UEBMI and URBMI, i.e. individual records were categorized into those with or without tagging text. For records with tagging text, two orthopedic surgeons reviewed independently to further exclude the inappropriate expression. For records without tagging text, the cosine distance was calculated. Two thresholds, i.e. 0.4 and 0.6 were used to classify individual records. If the cosine similarity of all the diagnostic terms is less than 0.4, corresponding records were discarded. If the cosine similarity is between 0.4 and 0.6, the corresponding records were reviewed by two orthopedic surgeons independently to further include the potential missing patients with hip fracture. If we find any, the related records were tagged. Finally, 477,076 records from 190,560 patients were reviewed and included as case of hip fractures in our study.

**References**

1. Leslie WD, O'Donnell S, Jean S, Lagace C, Walsh P, Bancej C, et al. Trends in hip fracture rates in Canada. JAMA. 2009;302(8):883-9.
2. Karayiannis PN, McAlinden MG. Falling age-related incidence of hip fractures in women, but not men, in Northern Ireland: 2001-2011. Osteoporos Int. 2016;27(11):3377-81.
3. Ha YC, Kim TY, Lee A, Lee YK, Kim HY, Kim JH, et al. Current trends and future projections of hip fracture in South Korea using nationwide claims data. Osteoporos Int. 2016;27(8):2603-9.
4. Azizieh FY. Incidence of hip fracture in Kuwait: a national registry-based study. Arch Osteoporos. 2015;10:40-6.
5. Brauer CA, Coca-Perraillon M, Cutler DM, Rosen AB. Incidence and mortality of hip fractures in the United States. JAMA. 2009;302(14):1573-9.

**Table A1 Keywords used to search for extracting hip fracture cases.**

| **Diagnostic name** | **Chinese characters** | **Corresponding English translation** | **ICD-9** | **ICD-10** |
| --- | --- | --- | --- | --- |
| Hip fracture | 髋部骨折 | Hip fracture | 820 | S72.0/72.1 |
| Femoral neck fracture | 股骨颈骨折 | femoral neck fracture | 820 | S72.0 |
| Femoral neck fracture | 股骨颈囊内骨折 | intracapsular fracture of the femoral neck | 820 | S72.0 |
| Femoral neck fracture | 头下型股骨颈骨折 | subcapital femoral neck fracture | 820 | S72.0 |
| Femoral neck fracture | 股骨颈头下型骨折 | subcapital fracture of femoral neck | 820 | S72.0 |
| Femoral neck fracture | 股骨颈骨折(头下型) | femoral neck fracture (subcapital) | 820 | S72.0 |
| Femoral neck fracture | 基底型（部）股骨颈骨折 | basicervical femoral neck fracture | 820 | S72.0 |
| Femoral neck fracture | 股骨颈基底型（部）骨折 | basicervical fracture of femoral neck | 820 | S72.0 |
| Femoral neck fracture | 股骨颈骨折（基底型） | femoral neck fracture (basicervical) | 820 | S72.0 |
| Femoral neck fracture | 头颈型股骨颈骨折 | transcervical femoral neck fracture | 820 | S72.0 |
| Femoral neck fracture | 经颈型股骨颈骨折 | transcervical femoral neck fracture | 820 | S72.0 |
| Femoral neck fracture | 股骨颈经颈型骨折 | transcervical fracture of femoral neck | 820 | S72.0 |
| Femoral neck fracture | 股骨颈头颈型骨折 | transcervical fracture of femoral neck | 820 | S72.0 |
| Femoral neck fracture | 股骨颈骨折（经颈型） | femoral neck fracture (transcervical) | 820 | S72.0 |
| Femoral neck fracture | 股骨颈骨折（头颈型） | femoral neck fracture (transcervical) | 820 | S72.0 |
| [Intertrochanteric](javascript:;) femur [fracture](javascript:;) | 粗隆间骨折 | [intertrochanteric](javascript:;) [fracture](javascript:;) | 820 | S72.1 |
| [Intertrochanteric](javascript:;) femur [fracture](javascript:;) | 股骨逆粗隆间骨折 | reverse obliquity intertrochanteric femur fracture | 820 | S72.1 |
| [Intertrochanteric](javascript:;) femur [fracture](javascript:;) | 逆粗隆间骨折 | reverse obliquity intertrochanteric fracture | 820 | S72.1 |
| [Intertrochanteric](javascript:;) femur [fracture](javascript:;) | 转子间骨折 | [intertrochanteric](javascript:;) [fracture](javascript:;) | 820 | S72.1 |
| [Intertrochanteric](javascript:;) femur [fracture](javascript:;) | 股骨逆转子间骨折 | reverse obliquity intertrochanteric femur fracture | 820 | S72.1 |
| [Intertrochanteric](javascript:;) femur [fracture](javascript:;) | 逆转子间骨折 | reverse obliquity intertrochanteric fracture | 820 | S72.1 |
| **Special symptoms and sign** | | | | |
| Hip pain | 髋部疼痛 | hip pain |  |  |
| Shortening and external rotation of the affected leg | 患肢短缩外旋畸形 | shortening and external rotation of the affected leg |  |  |
| **Special examination and treatment** | | | | |
| Hip X- ray | 髋部X线 | hip X- ray |  |  |
| Open reduction and internal fixation of femoral neck fracture | 股骨颈骨折切开复位内固定 | open reduction and internal fixation of femoral neck fracture |  |  |
| Open reduction and internal fixation of intertrochanteric fracture | 股骨粗隆间骨折切开复位内固定 | open reduction and internal fixation of intertrochanteric fracture |  |  |

**Figure A1 Flow chart of case ascertainment.**

**
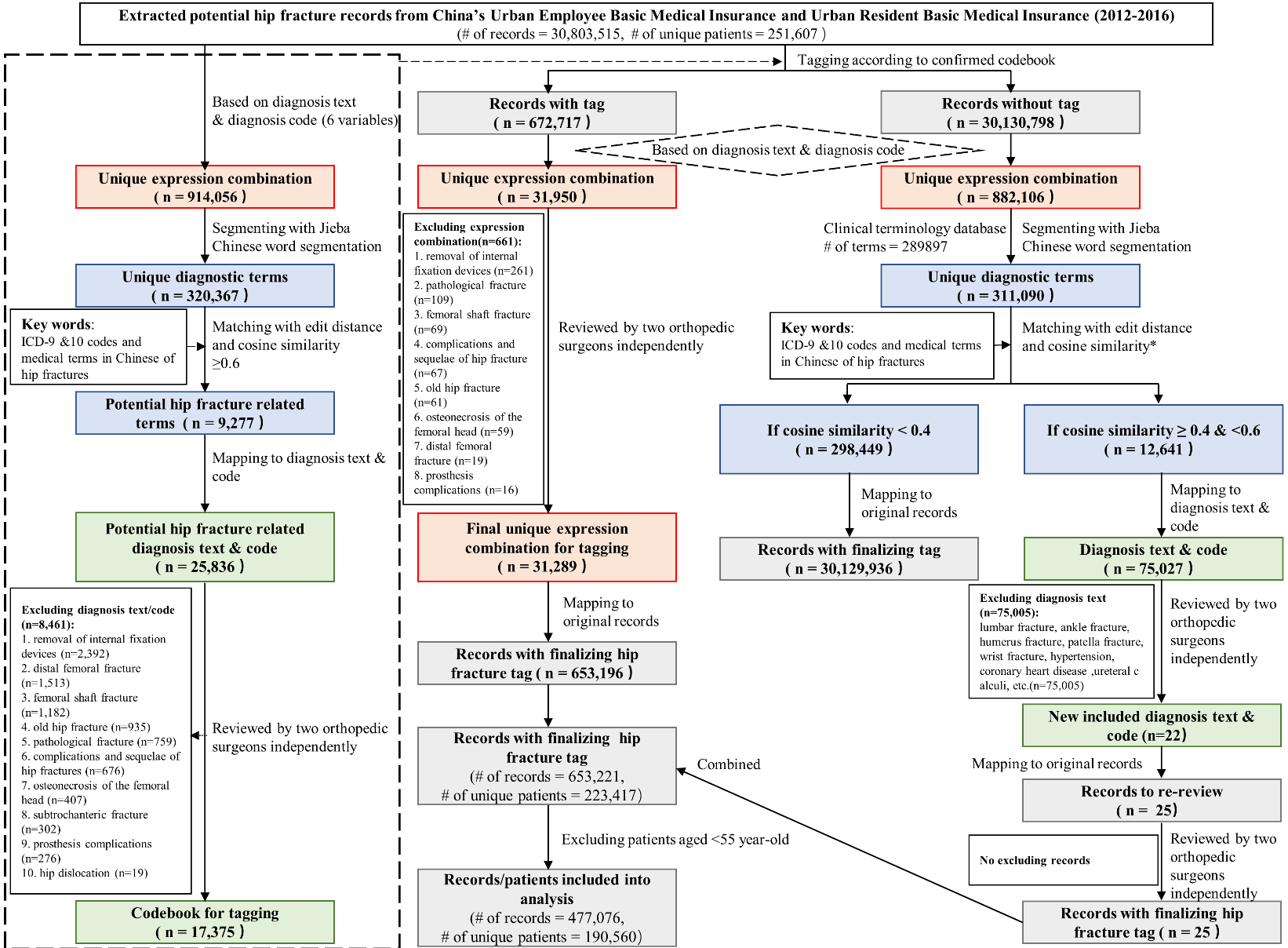
**

Note: If the cosine similarity of all the diagnostic terms is less than 0.4, corresponding records were discarded. If the cosine similarity is between 0.4 and 0.6, the corresponding records were reviewed by two orthopedic surgeons independently to further include the potential missing patients with hip fracture.

1. **Statistical method to estimate the incidence of hip fracture**

Incidence of hip fracture was estimated by a two-stage approach.

1. **Stage 1: Estimation of age- and sex-specific incidence in each province**

The denominator used to calculate the annual incidence of hip fracture is defined as the total number of individuals in the UEBMI and URBMI in each provincial age and sex-specific groups during the year. In the pilot analysis for the national insurance database, we found that the diagnostic information may be missing, often due to administrative reasons. The total enrolled population in each subgroup (*N_ij_*) can be classified into three categories: individuals with no records of any medical claim (*N_1ij_*), individuals with complete information on diagnosis in claim records (*N_2ij_*), and individuals with claim records but missing diagnostic information (*N_3ij_*). Complete-case analysis was not feasible in this case due to the existence of the population without any claim (i.e. healthy population which accounts for a significant proportion of the population). Ignoring the missingness will lead to the underestimation of the incidence. Therefore, we designed the following imputation algorithm to take account for the influence caused by missing information on the diagnosis.

The total population can be divided as shown in the following table.

|  | **Case** | **Non-case** | **Total** |
| --- | --- | --- | --- |
| Individuals with diagnostic information | *a_ij_* | *b_ij_* | *N_2ij_* |
| Individuals without diagnostic information | *c_ij_* | *d_ij_* | *N_3ij_* |
| Individuals without any claim | 0 | *e_ij_* | *N_1ij_* |
| **Population in each subgroup** | ***M_ij_*** | ***N_ij_ - M_ij_*** | ***N_ij_*** |

where *i* denoted the age- and sex- subgroup and *j* represented the province. Patients with hip fracture, noted as *M_ij_*, is referred to the nominator to calculate the incidence. Incidence can then be calculated as

$$\boldsymbol{I}_{\boldsymbol{ij}}=\frac{\boldsymbol{a}_{\boldsymbol{ij}}+\boldsymbol{c}_{\boldsymbol{ij}}}{\boldsymbol{a}_{\boldsymbol{ij}}+\boldsymbol{b}_{\boldsymbol{ij}}+\boldsymbol{c}_{\boldsymbol{ij}}+\boldsymbol{d}_{\boldsymbol{ij}}+\boldsymbol{e}_{\boldsymbol{ij}}} (A2)$$

Considering the fact that the missingness of diagnosis was often due to administrative reasons which should not be specific to the disease of hip fracture, we assumed the proportion of hip fractures cases in individuals with or without diagnostic information were equal, i.e.

$$\frac{\boldsymbol{a}_{\boldsymbol{ij}}}{\boldsymbol{a}_{\boldsymbol{ij}}+\boldsymbol{b}_{\boldsymbol{ij}}}=\frac{\boldsymbol{c}_{\boldsymbol{ij}}}{\boldsymbol{c}_{\boldsymbol{ij}}+\boldsymbol{d}_{\boldsymbol{ij}}} (A3)$$

The number of cases in individuals without diagnostic information (*c_ij_*) was then expressed as

$$\boldsymbol{c}_{\boldsymbol{ij}}=\frac{\boldsymbol{a}}{N_{2ij}}\times N_{3ij}=r_{ij}N_{3ij} (A4)$$

where$r_{ij}$ was referred to the incidence rate in individuals with complete information on diagnosis. In each province, we fit the Poisson regression model as

$r_{j}= e^{\beta_{0}+\beta_{1}\text{Age}+\beta_{1}\text{Sex}+\beta_{2}\text{Year+}\beta_{2}\text{Type}}$ (A5)

where *Type* was referred to the type of the insurance (i.e. UEBMI or URBMI). The expected number of cases in each subgroup (including both subjects with and without diagnostic information) can then be calculated based on the predictions from the Poisson model. Moreover, to account for the uncertainty from the model prediction, we generated 10 estimated number of cases sampled from the distribution. For the crude analysis, the province-specific incidence was directly calculated using the equation A2 where each element was the summation of all age- and sex- subgroups, e.g. $a_{j}=\sum_{i} a_{ij}$. Finally, the incidence for each province or subgroup was calculated by pooling ten estimates using Rubin’s Rule.

1. **Stage 2: Pooling at the national level and Standardization**

In the second stage, for the crude analysis, the province-specific incidences were pooled using a random-effects meta-analysis. For the adjusted analysis, the estimate of the national incidence in each age- and sex- subgroup is first calculated by combining province-specific estimates using a random-effects meta-analysis. The overall national estimate of the incidence was then standardized as a weighted average according to China 2010 census data.

1. **Pre-specified Statistical Analysis Plan (SAP)**
2. **Study objective:**

To estimate the incidence of hip fracture and associated costs for hospitalization in hip fracture patients aged 55 years and older in China by using the national insurance database using a cohort study design.

1. **Database and study period**

- Chinese Urban Employee Basic Medical Insurance (UEBMI) database: 01/01/2012-31/12/2016
- Chinese Urban Resident Basic Medical Insurance (URBMI) database: 01/01/2012-31/12/2016

1. **Outcome**

The outcomes to be investigated in our study include:

- Incidence of hip fracture.
- Associated costs for hospitalization including total costs per year and costs per patient per year.

Hospital costs include costs associated with surgery, anesthesia, medical materials, imaging investigations and pharmaceuticals. Costs are discounted by the consumer price index (CPI) in each year to 2016 costs and convert into US dollars based on the 2016 RMB to US dollar exchange rate (period average). The CPI and exchange rate are from China Statistical Yearbook 2017.

1. **Other covariates definition**

- Age: continuous and categorical variable defined as 55-64, 65-74, 75-84 and ≥85 years old.
- Sex (men, women)
- Insurance type (URBMI, UEBMI)
- Year (2012, 2013, 2014, 2015, 2016)

1. **Methods**
2. **Incidence**

Crude and adjusted incidence of hip fracture are calculated. Incidence of hip fracture is estimated by a two-stage approach. (Note: detailed description for estimating the incidence in the primary analysis is moved to the Section II from the original SAP).

1. **Cost for hospitalization**

Total cost for hospitalization is calculated as the sum of the annual hospital costs of patients. Hospital costs per patient per year is the total hospital cost divided by the expected number of patients in the corresponding year.

1. **Primary statistics to report:**

Incidence is expressed as per 100,000 person-years at risk, and 95% confidence intervals (CIs). Hospital costs are discounted by consumer price index (CPI) in each year to 2016 costs and expressed in US dollars.

1. **Subgroup analysis**

Incidence will be calculated in subgroups of calendar year, sex, age group and sex-age groups.

1. **Sensitivity analysis**

The following sensitivity analyses will be conducted:

- Including only observed cases to investigate the lower bounds of the rates
- Excluding the top 10% of provinces ranked by the missingness of diagnostic information.

1. **Proposed Main Tables & Figures**

**Table 1 Characteristics for hip fracture patients grouped by sex.**

- Total number of patients and sex-specific number of patients
- Median and mean age of patients and sex-specific median age of patients
- Total number of patients in age groups and sex-specific number of patients in age groups
- Total number of patients in each year and sex-specific number of patients in each year
- Total number of patients in different ethnicities and sex-specific number of patients in different ethnicities
- Total number of patients in different areas and sex-specific number of patients in different areas

**Table 2 Crude incidence of hip fractures grouped by sex and age group.**

- Crude incidence in each year during 2012~2016
- Crude incidence grouped by sex in each year during 2012~2016
- Crude incidence grouped by age group in each year during 2012~2016

**Table 3 List of Provinces in China included in the study**

- 23 provinces over the period from January 1st, 2012 to December 31th, 2016 in the UEBMI and URMBI
- Area distribution of the 23 provinces

**Table 4 Basic characteristics of population aged 55 years and older in 23 provinces of China during 2012–2016**

- Total number of population and insurance-specific number of population
- Median and mean age of population and insurance-specific median age of population
- Total number of population in age groups and insurance-specific number of population in age groups
- Total number of population in sex groups and insurance-specific number of population in sex groups
- Total number of population in different ethnicities and insurance-specific number of population in different ethnicities
- Total number of population in different areas and insurance-specific number of population in different areas

**Table 5 Results of the sensitivity analysis**

- Including only observed cases to assess the lower bounds of the rates
- Excluding the top 10% of provinces with missing diagnostic information.

**Table 6 Adjusted incidence of hip fracture.**

- Adjusted incidence in each year during 2012~2016 using China 2010 census data
- Adjusted incidence grouped by sex in each year during 2012~2016

**Figure 1 Crude incidence, adjusted incidence and annual number of hip fractures.**

- Crude incidence in each year during 2012~2016
- Adjusted incidence in each year during 2012~2016 using China 2010 census data
- Annual number of hip fracture patients in each year during 2012~2016

**Figure 2 Total costs, costs per patient and annual number of hip fractures.**

- Total hospital costs in each year during 2012~2016
- Hospital costs per patient in each year during 2012~2016
- Annual number of hip fracture patients in each year during 2012~2016

**Figure 3 Crude incidence of hip fracture in population grouped by sex, age and sex–age group.**

- Crude incidence grouped by sex in each year during 2012~2016
- Crude incidence grouped by age group in each year during 2012~2016
- Crude incidence grouped by sex-age group in each year during 2012~2016

**Figure 4 Changes in median and mean age of hip fracture patients.**

- Median age of hip fracture patients in each year during 2012~2016
- Mean age of hip fracture patients in each year during 2012~2016
