# Supplementary figures and images for: Incidence of and trends in hip fracture among adults in urban China: A nationwide retrospective cohort study
Source: PLoS Med. 2020 Aug 6;17(8):e1003180. doi: 10.1371/journal.pmed.1003180 (PMC7410202; doi:10.1371/journal.pmed.1003180)

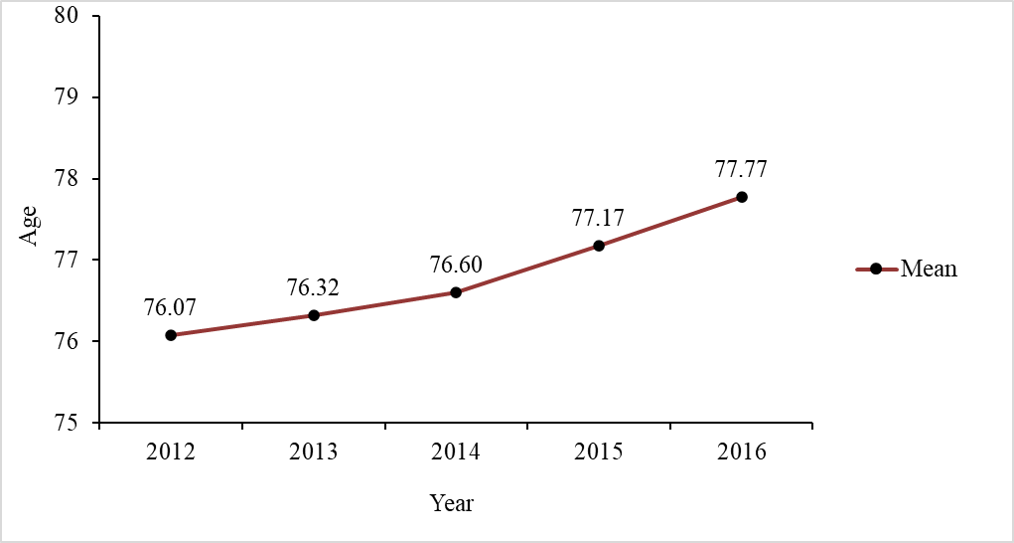

Supplement: S1 Fig — (TIF) [file pmed.1003180.s002.tif]
