# Supplementary material for: Incidence of and trends in hip fracture among adults in urban China: A nationwide retrospective cohort study
Source: PLoS Med. 2020 Aug 6;17(8):e1003180. doi: 10.1371/journal.pmed.1003180 (PMC7410202; doi:10.1371/journal.pmed.1003180)
Supplement: S1 Table — (DOCX) [file pmed.1003180.s004.docx]

## S1 Table. Basic characteristics of the population aged 55 years and older in 23 provinces of China during 2012–2016.

|  | | **Total** | **UEBMI** | **URBMI** |
| --- | --- | --- | --- | --- |
|  |  |  |  |  |
| Total Number, n (%) |  | 102.56 (100.00) | 47.29 (100.00) | 55.27 (100.00) |
| Age, y | Mean (SD) | 67.61 (9.54) | 66.97 (9.29) | 68.16 (9.72) |
| Age group, n (%) | 55-64 | 48.08 (46.88) | 23.65 (50.02) | 24.43 (44.20) |
|  | 65-74 | 30.55 (29.79) | 13.19 (27.90) | 17.36 (31.41) |
|  | 75-84 | 17.45 (17.01) | 7.97 (16.85) | 9.48 (17.15) |
|  | ≥85 | 6.48 (6.32) | 2.47 (5.23) | 4.01 (7.25) |
| Gender, n (%) | Male | 52.83 (51.51) | 28.06 (59.33) | 24.77 (44.81) |
|  | Female | 49.73 (48.49) | 19.23 (40.67) | 30.50 (55.19) |
| Ethnicity, n (%) | Han | 87.95 (85.75) | 43.05 (91.04) | 44.90 (81.23) |
|  | Others | 3.96 (3.86) | 1.82 (3.84) | 2.14 (3.87) |
|  | Unknown | 10.66 (10.49) | 2.42 (5.12) | 8.24 (14.90) |
| Area, n (%) | East | 42.37 (41.31) | 14.82 (31.33) | 27.55 (49.85) |
|  | North | 4.40 (4.29) | 2.74 (5.78) | 1.66 (3.01) |
|  | North-East | 14.95 (14.58) | 10.77 (22.77) | 4.18 (7.57) |
|  | North-West | 4.65 (4.53) | 2.96 (6.26) | 1.69 (3.06) |
|  | South-Central | 21.83 (21.28) | 11.59 (24.50) | 10.24 (18.52) |
|  | South-West | 14.37 (14.01) | 4.42 (9.35) | 9.95 (17.99) |

Data presented as millions of individuals (percent).

Abbreviations: UEBMI, Urban Employee Basic Medical Insurance; URBMI, Urban Resident Basic Medical Insurance. SD, standard deviation.

East area included Jiangsu, Zhejiang, Anhui, Jiangxi and Shandong provinces; North area included Shanxi and Inner Mongolia provinces; North-East area included Liaoning, Jilin and Heilongjiang provinces; North-West area included Shaanxi, Gansu, Qinghai and Xinjiang provinces; South-Central area included Henan, Hubei, Hunan, Guangdong, Guangxi and Hainan provinces; South-West area included Chongqing, Guizhou and Yunnan provinces.
