# Supplementary material for: Incidence of and trends in hip fracture among adults in urban China: A nationwide retrospective cohort study
Source: PLoS Med. 2020 Aug 6;17(8):e1003180. doi: 10.1371/journal.pmed.1003180 (PMC7410202; doi:10.1371/journal.pmed.1003180)
Supplement: S2 Table — (DOCX) [file pmed.1003180.s005.docx]

## S2 Table. Sensitivity analysis of hip fracture incidence using only observed cases or excluding the top 10% of provinces ranked by the missingness of diagnostic information (unit:/100,000 person-year).

|  | 2012 | 2013 | 2014 | 2015 | 2016 |
| --- | --- | --- | --- | --- | --- |
| Using only observed cases | 80.47  (60.96,99.98) | 80.11  (61.56,98.66) | 77.42  (59.69,95.15) | 74.42  (51.07,97.76) | 96.27  (68.92,123.63) |
| Excluding the top 10% of provinces with missing diagnostic information ^a^ | 149.71  (113.99,185.44) | 174.891  (141.72,208.06) | 150.22  (122.04,178.41) | 145.03  (118.54,171.53) | 147.90  (119.80,176.00) |

a: Shandong and Jiangxi provinces were excluded; the above table cells were the incidences and their 95% confidence intervals.
