# Supplementary material for: Incidence of and trends in hip fracture among adults in urban China: A nationwide retrospective cohort study
Source: PLoS Med. 2020 Aug 6;17(8):e1003180. doi: 10.1371/journal.pmed.1003180 (PMC7410202; doi:10.1371/journal.pmed.1003180)
Supplement: S3 Table — Standardized by the population from the China 2010 census data. (DOCX) [file pmed.1003180.s006.docx]

## S3 Table. Adjusted incidence of hip fractures (units: /100,000 person-year).

|  | **Adjusted incidence (95% confidence interval)** | | |
| --- | --- | --- | --- |
|  | **Total** | **Male** | **Female** |
| 2012 | 128.10 (88.68-174.79) | 108.00 (72.84-150.07) | 152.17 (105.70-207.12) |
| 2013 | 138.43 (104.04-177.74) | 108.42 (80.74-140.19) | 165.54 (123.26-214.06) |
| 2014 | 117.10 (90.40-147.27) | 99.12 (76.60-124.55) | 137.36 (105.18-173.86) |
| 2015 | 106.02 (81.56-133.69) | 86.47 (66.18-109.47) | 125.62 (95.36-160.04) |
| 2016 | 114.46 (89.85-142.06) | 88.01 (70.17-107.88) | 142.14 (110.31-178.05) |

Standardized by the population from the China 2010 census data.
