# Supplementary material for: Incidence of and trends in hip fracture among adults in urban China: A nationwide retrospective cohort study
Source: PLoS Med. 2020 Aug 6;17(8):e1003180. doi: 10.1371/journal.pmed.1003180 (PMC7410202; doi:10.1371/journal.pmed.1003180)
Supplement: Attachment — Submitted filename: Response to Editors and Reviewers.docx [file pmed.1003180.s007.docx]

**Responses to comments of editors and reviewers**

***Requests from the editors:***

1. Please revise your title according to PLOS Medicine's style. Your title must be nondeclarative and not a question. It should begin with main concept if possible. "Effect of" should be used only if causality can be inferred, i.e., for an RCT. Please place the study design ("A randomized controlled trial," "A retrospective study," "A modelling study," etc.) in the subtitle (ie, after a colon).

*Response*

As requested, we revised the full title as “Incidence of and trends in hip fracture among adults in urban China: a nationwide retrospective cohort study.”

2. The Data Availability Statement (DAS) requires revision. For each data source used in your study:

a) If the data are freely or publicly available, note this and state the location of the data: within the paper, in Supporting Information files, or in a public repository (include the DOI or accession number).

b) If the data are owned by a third party but freely available upon request, please note this and state the owner of the data set and contact information for data requests (web or email address). Note that a study author cannot be the contact person for the data.

c) If the data are not freely available, please describe briefly the ethical, legal, or contractual restriction that prevents you from sharing it. Please also include an appropriate contact (web or email address) for inquiries (again, this cannot be a study author).

*Response*

As requested, we included the contact email in the revised version (page 20, line 425-427).

3. Abstract

Last sentence of methods and findings should highlight a limitation of the study methodology/study design

*Response*

We added a limitation of study into the “Methods and Findings” section in the abstract as “the main limitation of the study was the unavailability of data on imaging information to adjudicate cases of hip fracture” (page 3, line 50-51).

Please provide brief demographics of the cohort

*Response*

In the revised abstract, we described the brief demographics of the cohort as “A total of 190,560 incident hip fracture patients (mean age 77.05 years, standard deviation 8.94; 63.99% female) were identified” (page 2, line 36-38).

4. Conclusion

Conclusions- unclear how hip fractures over 55 are a surrogate for osteoporotic fractures. Please clarify and revise as needed

*Response*

We thank the editor for this comment. It is known that patients aged 55 years and above are more likely to have sustained hip fractures because of osteoporosis with a low impact energy trauma (generally arising from a fall) **(*Osteoporos Int.*** 2008; 19:1267-73; [***Maturitas***](https://www.ncbi.nlm.nih.gov/pubmed/26255681)***.*** 2015; 82:85-93; ***Osteoporos Int.*** 2014; 25:2359–2381; [***Osteoporos Int***](https://www.ncbi.nlm.nih.gov/pubmed/17641811)***.*** 2008; 19:79-86;). Younger patients probably suﬀered from high impact energy trauma resulting in hip fracture not related to osteoporosis **(*Osteoporos Int.*** 2008; 19:1267-73; [***Maturitas***](https://www.ncbi.nlm.nih.gov/pubmed/26255681)***.*** 2015; 82:85-93; ***Osteoporos Int.*** 2014; 25:2359–2381; [***Osteoporos Int***](https://www.ncbi.nlm.nih.gov/pubmed/17641811)***.*** 2008; 19:79-86;). In addition, indicators of osteoporosis such as bone density or imaging information are seldom available in the medical insurance database. Therefore, researchers generally used hip fractures over 55 as a surrogate for osteoporotic fractures. This is generally accepted and consistent with previous publications on hip fracture incidence (***JAMA.*** 2009; 302:1573-9; ***JAMA.*** 2009; 302:883-889; ***J Bone Miner Res. 2***012; 27:125-9; ***Bone.*** 2015; 81:131-137; ***J Clin Med.*** 2019; 8:1487-1505). In this revision, to avoid over-interpretation, we removed the inference to the osteoporotic fractures in the conclusion (Page 3, line 52-55) but kept the statements above in Method in Page 7, line 138-146 and relative discussion in Page 14, line 299-301.

* Please address the study implications without overreaching what can be concluded from the data; the phrase "In this study, we observed ..." may be useful.

* Please interpret the study based on the results presented in the abstract, emphasizing what is new without overstating your conclusions.

* Please avoid vague statements such as "these results have major implications for policy/clinical care". Mention only specific implications substantiated by the results.

* Please avoid assertions of primacy ("We report for the first time....")

*Response*

We thank the Editors for this comment. We have rewritten the “Conclusion” section as “Hip fracture incidence among patients aged 55 and over in China had reached a plateau between 2012 and 2016. However, the absolute number of hip fracture and associated medical costs for hospitalization increased rapidly because of population aging (Page 3, line 52-55).

5. Author summary

Please use an alternative for “most developed countries”. Do you mean specific countries or high-income nations?

*Response*

We have replaced “developed countries” with “high-income nations” throughout the revised manuscript (page 5, line 90-92,94-95; page 13, line 284; page 14, line 287,297).

I’m not sure this can be directly inferred from your data so please revise “Our findings indicated that strategies for osteoporosis prevention and therapy may have been effective in China over the past few years”.

*Response*

We thank the Reviewer for this valuable comment. Studies in the United States, and Hong Kong suggested that the number of anti-osteoporosis treatments may be associated with the decline in hip fracture incidence (***JAMA.*** 2009;302(14):1573-9; ***Age Ageing.*** 2013;42(2):229-33), whereas a few other studies held a suspicious attitude as the decline appeared before the availability of use of bisphosphonate **(*JAMA.*** 2009;302(8):883-9; ***Osteoporos Int.*** 2014;25(1):205-10). So far it is not clear how much the use of osteoporotic medication account for the reduction of hip fracture incidence. In China, the guidelines and policies about osteoporosis prevention and therapy were both issued in 2011, which happened to be just earlier than the beginning of our study period. In addition, rapid increase use of anti-osteoporosis drugs was also reported in different regions between 2008 and 2012 in China (***Chinese Journal of Drug Application and Monitoring.*** 2011;8(6):372-4; ***China Pharmacy.*** 2014;25(22):2029-31), along with the market release of zoledronic acid (2009) and teriparatide (2012). Compared with the hip fracture incidence during 2002-2006 in Beijing, China (229 per 100,000 person-years in females and 129 per 100,000 person-years in males, ***Journal of Bone & Mineral Research.*** 2012; 27:125-129.), the hip fracture incidence in our study was lower in both females and males, especially in 2016. Therefore, we speculated that the wide prescription of anti-osteoporotic drugs might have an important impact on osteoporosis, and resulted in this plateau period of hip fracture incidence. However, we acknowledged that due to missing information on individual osteoporotic drugs usage, we cannot draw the direct causal relationship and therefore further studies are still needed. Therefore, to avoid over-interpretation, we deleted this from the author summary.

Please rephrase “unparallel trends” to simpler and more accessible language.

*Response*

The sentence containing “unparallel trends” was deleted in this revision.

6. Introduction

Since you cite previous studies on a similar topic, and note that they evaluated costs over a short time interval, please comment on why 3 years is considered a short time period but 4 (as in this case) isn’t?

*Response*

The period of our study was 5 years, starting from January 1st, 2012 to December 31th, 2016. In this revision, we rephrased the sentence as “often within short time intervals”. Furthermore, we clarified in the “Methods” section as “data were analyzed from a nationally representative population covering approximately 100 million residents aged 55 years and older in 23 provinces over the five-year period from January 1st, 2012 to December 31th, 2016.” (page 7, line 135-137).

Please address past research and explain the need for and potential importance of your study. Indicate whether your study is novel and how you determined that. If there has been a systematic review of the evidence related to your study (or you have conducted one), please refer to and reference that review and indicate whether it supports the need for your study.

*Response*

We thank the Editors for this helpful comment. We now clarified in the introduction as “in the past decade, the incidence of hip fractures in high-income nations has declined or reached a plateau after an initial increase (***JAMA.*** 2009;302(14):1573-9; ***JAMA.*** 2009;302(8):883-9; ***Arch Gerontol Geriatr.*** 2018;77:64-7; ***Bone.*** 2015;81:131-7; ***Bone.*** 2018;108:1-9; ***Bone.*** 2015;78:55-61. ). Compared with the vast majority of published studies conducted in high-income nations, the data is scarce in low or intermediate-income nations (***Arch Osteoporos.*** 2017;12(1):107-12; ***Arch Osteoporos.*** 2015;10:40-6; ***J Bone Miner Res.*** 2012;27(1):125-9). A systematic review conducted in 2014, including 22 studies from high-income nations/regions and 3 from low or middle-income nations, indicated that hip fracture incidence in high-income nations/regions began to decrease after an initial increase, but still increased rapidly in low or middle-income nations (***J Bone Miner Res.*** 2014;29(8):1745-55). ……China, the largest low or intermediate-income nation, has the largest population in the world and has been experiencing unprecedentedly rapid population aging. ……. However, only a few studies have evaluated trends in hip fracture incidence and hospital costs in China, using relatively small samples, often within short time intervals or in several regional areas.” (page 5-6, line 90-97,105-108)

7. Methods

Please ensure that the study is reported according to the STROBE guideline. Please add the following statement, or similar, to the Methods: "This study is reported as per the Strengthening the Reporting of Observational Studies in Epidemiology (STROBE) guideline (SI file xxx)."

*Response*

We thank the Editors for this comment. As requested, we have added this as S2 STROBE checklist in the revised manuscript. (page 11, line 223-224).

8. Did your study have a prospective protocol or analysis plan? Please state this (either way) early in the Methods section.

a) If a prospective analysis plan (from your funding proposal, IRB or other ethics committee submission, study protocol, or other planning document written before analyzing the data) was used in designing the study, please include the relevant prospectively written document with your revised manuscript as a Supporting Information file to be published alongside your study, and cite it in the Methods section. A legend for this file should be included at the end of your manuscript.

b) If no such document exists, please make sure that the Methods section transparently describes when analyses were planned, and when/why any data-driven changes to analyses took place.

c) In either case, changes in the analysis-- including those made in response to peer review comments-- should be identified as such in the Methods section of the paper, with rationale.

*Response*

We thank the Editors for this comment. We translated the prospective analysis plan into English and added it as S1 Appendix (Section III).

9. Please clarify why the time period 2012-2016 was chosen?

*Response*

We thank the Editors for this comment. China achieved universal health insurance coverage in 2011. The time period was chosen only due to the availability of our access to the databases for UEBMI and URBMI We now clarified in method section (page 7, line 137-138).

10. Please describe the inclusion and exclusion criteria in full- including age/co-morbidities etc.

*Response*

We thank the Editors for this comment. We have now clearly described the inclusion and exclusion criteria of the study population in the Method (page 7-8, line 146-152,157-160; page 9, line 171-180).

11. Please clarify how you determined the age cut off of 55 years?

*Response*

We thank the Editors for this comment. We now clarified in the Method as “it is known that patients aged 55 years and above are more likely to have sustained hip fractures because of osteoporosis with a low impact energy trauma (generally arising from a fall) (***Osteoporos Int.*** 2008; 19:1267-73; [***Maturitas***](https://www.ncbi.nlm.nih.gov/pubmed/26255681)***.*** 2015; 82:85-93; ***Osteoporos Int.*** 2014; 25:2359–2381; [***Osteoporos Int***](https://www.ncbi.nlm.nih.gov/pubmed/17641811)***.*** 2008; 19:79-86). Younger patients probably suﬀered from high impact energy trauma resulting in hip fracture not related to osteoporosis (***Osteoporos Int.*** 2008; 19:1267-73; [***Maturitas***](https://www.ncbi.nlm.nih.gov/pubmed/26255681)***.*** 2015; 82:85-93; ***Osteoporos Int.*** 2014; 25:2359–2381; [***Osteoporos Int***](https://www.ncbi.nlm.nih.gov/pubmed/17641811)***.*** 2008; 19:79-86). In addition, indicators of osteoporosis such as bone density or imaging information are seldom available in the medical insurance database. Therefore, consistent with previous publications on hip fracture incidence (***JAMA.*** 2009; 302:1573-9; ***JAMA.*** 2009; 302:883-889; ***J Bone Miner Res.*** 2012; 27:125-9; ***Bone.*** 2015; 81:131-137; ***J Clin Med.*** 2019; 8:1487-1505), in this study, hip fracture in patients aged 55 years and above were used as a surrogate for determining the conditions of osteoporosis or osteoporotic fractures.”.

12. Please describe the adjustments with China 2010 census data in the methods as noted in Fig 1. Same goes for the cost discounted using CPI, exchange rate etc in Fig 3

*Response:* DONE.

13. Please provide details of algorithm used and revise the term “relatively loose” to use more appropriate scientific language.

14. Please provide the keywords used (original language and English translation) as supplementary information such that the search may be replicated.

*Response*

We thank the Editors for this comment. The details of algorithm as well as the keywords (original language and English translation) were provided in the Section I of the appendix (S1 Appendix). Consistent with similar studies (***Database the Journal of Biological Databases & Curation.*** 2018, 1-10; ***Journal of Biomedical Informatics.*** 2016, 64:1-9), we replaced the term “relatively loose” with “fuzzy string matching” in the revised manuscript (page 8, line 161).

15. STROBE checklist- please remove page numbers as these are likely to change. Instead you may use paragraphs and sections.

*Response*: DONE.

16. Please revise “nearly decreasing trend” and use specific language instead.

*Response*

We thank the Editors for this comment. We deleted this expression in the revised manuscript.

17. Please provide p values along with 95% confidence intervals. Please note exact p values should be provided if p>0.05 or p<0.05, p<0.001 is permitted.

*Response*

We thank the Editors for this comment. As suggested by Reviewer 2, we deleted the p values for group comparisons in table 1, considering the huge sample size in this study. In the footnote of Table 2, for the we stated that incidence rates with 95% confidence intervals, all rates are significantly larger than 0 with p<0.001. Meanwhile, the linear trend test for either overall or subgroup incidences across five years were not significant (all *P*>0.05).

18. Discussion

Please present and organize the Discussion as follows: a short, clear summary of the article's findings; what the study adds to existing research and where and why the results may differ from previous research; strengths and limitations of the study; implications and next steps for research, clinical practice, and/or public policy; one-paragraph conclusion.

*Response*

We thank the Editors for this helpful comment. We re-organized the “Discussion” section as suggested in the revised manuscript (page 13, line 272-277; page 14, line 299-302; page 15, line 309-312,315-330).

19. Please clarify if zoledronic acid is provided to those at risk of osteoporosis or is available over the counter etc.

*Response*

We thank the Editors for this comment. We now clarified in the manuscript (page 15, line 309-312) that zoledronic acid was generally available only by prescription for postmenopausal women as the first-line treatment to prevent osteoporosis and reduce the risk of hip, vertebral, and other fractures ([***N Engl J Med***.](https://www.ncbi.nlm.nih.gov/pubmed/17476007/) 2007, 356(18):1809-22). It is not sold over the counter in China and is available only by prescription.

***Comments from the reviewers:***

***Reviewer #1***

Overall, this is a well-written and interesting manuscript describing the incidence and costs of hip fractures in China. There are only a few minor points that could be clarified prior to publication:

1. The English language is very good, there are just a few minor grammatical issues which can be edited during the production process

*Response*.

We deeply appreciate your recognition of our research value and writing.

2. The sentence "Further decline is needed to reduce the absolute number of fractures and socioeconomic

burden resulting from population aging" is a clear conclusion. It would be better if the similar sentences in the Abstract and Conclusion could be replaced by this sentence instead. i.e. use this sentence instead of "Further decline is needed to reduce the heavy burden imposed by hip fracture".

*Response*.

We thank the Reviewer for this helpful comment. We have revised the manuscript in the Conclusion section accordingly (page 3, line 52-55).

3. In the Introduction section, it would be useful to provide a reference for the sentence "It is projected that the number of hip fractures worldwide…"

*Response*: DONE. A reference was added following the reviewer’s suggestion (page 5, line 88-90).

4. It would be useful to move the sentence "To reduce double-counting, subsequent fractures were

considered…" to the section titled "Hip Fracture Identification."

*Response*: DONE.

5. In the Results section, it could be useful to include a paragraph describing the absolute number of fractures.

*Response*.

We thank the Reviewer for this helpful comment. We have added a paragraph to describe the absolute number of fractures in the revised manuscript as “The overall number of hip fractures were 16,587, 27,727, 35,968, 43,703, and 66,575 for the years 2012, 2013, 2014, 2015, and 2016, respectively (table 1 and fig 3A). The mean age at the first hip fracture rose from 76.07(Standard deviation, SD: 9.43) to 77.77(SD: 8.66) during the 5-year period (P<0.001, S1 Fig),the proportion of females was 62.89% in 2012, and increasing to 65.26% in 2016 (*P*<0.001).” (page 12, line 251-256).

6. In the Discussion section, could the authors discuss the reasons that the costs per patient for hip fracture

have increased? The authors have provided an excellent discussion regarding the number and rates of hip

fractures, if they could also discuss costs per patient, that would strengthen the Discussion further.

*Response*.

We thank the Reviewer for this valuable comment. In this revision, we firstly clarified in the Method (Page 10, line 210-214) that the cost for hospitalization per patient in our study included those associated with surgery, anesthesia, medical materials, radiological investigations and pharmaceuticals. Then in the discussion (page 16-17, line 355-363), we stated that “As mentioned above, the Chinese guidelines and policies about osteoporosis prevention and therapy were both issued in 2011, which encouraged doctors to prescribe medical treatments to these patients. In addition, costs of inpatient drugs containing anti-osteoporosis medication were reported to account for 25.2% of the total direct costs for osteoporotic fractures (***Osteoporos Int.*** 2014;25(7):1853-60.). Along with the market release of zoledronic acid (2009) and teriparatide (2012), anti-osteoporosis drugs became more accessible. Rapid increase use of these drugs was also reported [24, 25]. Besides, the increases in costs of surgery and associated medical materials might also contribute.”

7. References 15 and 16 do not seem to be appropriate. It seems that these references were used because the authors used similar methods. It would be useful if the authors could find the original reference for that method. Otherwise, they should specify in the text that they completed their analyses using methods similar to these other studies.

*Response*.

We thank the Reviewer for this helpful comment. We have replaced them with the original reference (***Journal of epidemiology and community health.*** 2013;67(11):974-8.) for the statistical methods.

8. Reference 29 does not seem to be properly listed. Could the authors please check this?

*Response*: DONE

9. In Table 1 and Figure S1, the authors report both median and mean ages. If the data is normally distributed, the authors should report age as mean±SD. If not, then median (IQR). Could the authors please check their data and use either mean or median, but not both?

*Response*.

We thank the Reviewer for this helpful comment. Following the reviewer’s suggestion, we kept the mean results in both Table 1 and S1 Fig.

10. A map of the region would be useful, instead of a list of provinces (Table S1). Could the authors please provide a map, which will help international readers understand the region better?

*Response*: DONE. A map was provided as new Figure 1.

11. Could the authors please provide a more descriptive caption for Table S3?

*Response*.

We thank the Reviewer for this comment. To make the caption clearer, we have rewritten it as “Sensitivity analysis of hip fracture incidence using only observed cases or excluding the top 10% of provinces ranked by the missingness of diagnostic information (unit:/100,000 person-year)”.

***Reviewer #2***

I confine my remarks to statistical aspects of this paper. These were very well done, but I have a couple of minor points to address before I can recommend publication.

1. Overall, I would de-emphasize discussion of trends. The data in the table and figures don't show a linear trend and it's hard to see what trend they might show. It looks like noise.

*Response*.

We agreed that there was no significant decline trend, therefore chose the word of plateau to describe the results. As suggested, we have removed the statements about the decreasing trend. However, one message we would like to clearly deliver is that the trend of our study is distinct from the continuous rising trend reported in previous studies in low or middle-income nations (***J Bone Miner Res***. 2012 27:125-9. ***Arch Osteoporos***. 2017 12:107-12. ***Osteoporos***. 2015 10:40-6.), Therefore, we rephrased as “Incidence of hip fracture in China has stopped the continuous rising trend and entered a plateau period” in the author summary. We believe that this implication is very important for the prevention and control of hip fracture.

2. In Table 1, I would delete the p values - with N this large, everything is significant. And please list what staistic (t, chi square or whatever) is in the "Statistics" column

*Response:* DONE.

3. Figures 1 and 3 - I don't really like graphs with two axes - changing the limits of either axis changes the appearance a lot. Decide on what you want to show. If it's the relation between total cases and incidence/cost then a ratio could be plotted, or you can make a scatter plot of the two, with directed arrows showing trend over time. If that is not what you want to show, then either show only one set of data or adapt something to sut what you want to show.

*Response:* DONE.

***Reviewer #3:***

The methods are too brief to allow the reader to understand the results and require more detail.

*Response:* We thank the Reviewer for this comment. We have now provided a new Appendix to describe the details of our methods used as well as the pre-specified statistical analysis plan.

1. Case ascertainment is very important. More information is needed for the natural language processing in terms of training and test, internal / external validation. Were there changes in the quality of the text records over time.

*Response*.

We thank the Reviewer for this prudent comment. In this revision, we provided a new appendix as a supplementary document to describe the methods in detail (S1 Appendix). Flow-chart for case ascertainment was also presented. In addition, by reviewing the guidelines or clinical practice standards about the writing forms of the hip fractures in China, no evidence was found to indicate the potential changes in the quality of the text records during the study period.

2. What proportion of patients had surgery, did this change over time. Usually validation of cases requires

surgical / procedure codes. Were the types of patients identified by procedure codes different to those without.

*Response.*

We thank the Reviewer for this prudent comment. Unfortunately, surgical information was very limited and often incomplete in the databases, e.g. only total costs for all surgical treatments without detailed procedures or surgical codes were available. We addressed this in the limitation (page 18, line 383-385).

3. What is the definition of old hip fracture.

*Response*.

We thank the Reviewer for this comment. We now clarified in the Method that for case identification (page 9, line 177-180), patients were not included as a new case if (1) the fracture occurred within half a year after the previous one (if he/she had). This strategy is consistent with previous publications (***JAMA.*** 2009;302(14):1573-9); or (2) the diagnosis text was clearly described as “old hip fracture” by doctors.

4. How were cases really validated. It seems unfeasible for two surgeons to validate 190,560 cases.

*Response*.

We thank the Reviewer for this comment. Flow-chart and procedures for case ascertainment was provided in the Section I of the Appendix in this revision. The two orthopedic surgeons performed manual verification of the diagnostic text for all 190,560 cases with the assistance of the computer algorithm. As stated above, we clarified in the limitation that case ascertainment was limited due to missing information on laboratory, imaging or detailed surgical information (Page 18, line 383-385).

5. The numerator is described as taking into account missing data. What does this mean? Imputation?

missing data。

*Response*.

We thank the Reviewer for this comment. In order to avoid the underestimation of the incidence due to missing information on diagnosis, we used imputation method. Detail description about the method was provided in the Section II of the appendix (S1 Appendix).

6. The incidence seems to be per province population. I assume there was standardisation for both age and sex.

*Response*.

Clarified in the Section II of the appendix (S1 Appendix), in the adjusted analysis to get the national level incidences, the provincial age- and sex-specific rates were firstly calculated and then combined by meta-analysis to get corresponding rates at national level. Subsequently, the overall national rates were standardized with China 2010 census data. Alternatively, as the sensitivity analysis, subgroup-specific incidence rates can firstly standardized at the province level and then meta-analyzed to the national level. The standardized incidences using the alternative method were 144.05 (111.25, 176.86) per 100,000 person-years in 2012, 144.67 (114.08, 175.26) in 2013, 127.29 (102.20, 152.38) in 2014, 114.68 (91.90, 137.47) in 2015, and 121.43 (97.84, 145.03) in 2016, respectively. Results were very similar. We then chose to present the first method in the manuscript.

7. Is the reason for the decrease in incidence per population due to differences in age demographic over time.

*Response*.

We thank the Reviewer for this comment. First of all, we tried to control the potential effect of age with both standardization and stratification. All incidence rates for five years have been standardized using China 2010 census data for the national population. Meanwhile, the incidence rates were also estimated stratifying by gender (male, female) and age (55–64, 65–74, 75–84, and ≥ 85 years old). The results remained consistent. Furthermore, the incidence of hip fractures in fact increased with age. The proportions of the elderly aged 65 and above were slightly increasing from 2012 to 2016 according to Chinese national population statistics (from 9.39% to 10.85%). The aging effect of entire cohort should favor an increasing trend. However, we observed a stable (or even a declining) trend for the incidence in our study. Therefore, we don’t think the reason for the decrease in incidence was due to differences in age demographic over time.

8. It is not clear how costs were calculated and over what time period. This needs to be added to the methods

*Response*.

We thank the Reviewer for this helpful comment. We now clarified in the revision that the costs in our study included only inpatient costs, which were categorized as diagnosis related cost (including imaging investigations such as X-ray, Computerized tomography and Magnetic resonance imaging), surgical treatment (costs associated with surgery, anesthesia, medical materials) and pharmaceutical treatment (anti-osteoporosis drugs, etc.). We addressed this in the method (page 10, line 210-214)

9. What was the one year mortality, did it vary over time, sex and age group.

*Response*.

We thank the Reviewer for this comment. Unfortunately, death information was not available in these databases. We addressed this in the limitation (page 18, line 383-385).

10. Reductions in age specific hip fracture rates have been seen in other populations, often preceding DXA and pharmacological uptake in the community. Please comment

*Response*.

We thank the Reviewer for this valuable comment. Studies in the United States, and Hong Kong suggested that the number of anti-osteoporosis treatments may be associated with the decline in hip fracture incidence (***JAMA.*** 2009;302(14):1573-9; ***Age Ageing.*** 2013;42(2):229-33), whereas a few other studies held a suspicious attitude as the decline appeared before the availability of use of bisphosphonate **(*JAMA.*** 2009;302(8):883-9; ***Osteoporos Int.*** 2014;25(1):205-10). So far it is not clear how much percentage the use of osteoporotic medication account for the reduction of hip fracture incidence. In China, the guidelines and policies about osteoporosis prevention and therapy were both issued in 2011, which happened to be just earlier than the beginning of our study period. In addition, rapid increase use of anti-osteoporosis drugs was also reported in different regions between 2008 and 2012 in China (***Chinese Journal of Drug Application and Monitoring.*** 2011;8(6):372-4; ***China Pharmacy.*** 2014;25(22):2029-31), along with the market release of zoledronic acid (2009) and teriparatide (2012). Compared with the hip fracture incidence during 2002-2006 in Beijing, China (229 per 100,000 person-years in females and 129 per 100,000 person-years in males, ***Journal of Bone & Mineral Research.*** 2012; 27:125-129.), the hip fracture incidence in our study was lower in both females and males. Therefore, we speculated that the wide prescription of anti-osteoporotic drugs might have an important impact on osteoporosis, and resulted in this plateau period of hip fracture incidence. However, we acknowledged that due to missing information on individual osteoporotic drugs usage, we cannot draw the direct causal relationship and therefore further studies are still needed. We have now addressed this in the Discussion (page 15, line 315-330).
